# Comparative Ability of Mesenchymal Stromal Cells from Different Tissues to Limit Neutrophil Recruitment to Inflamed Endothelium

**DOI:** 10.1371/journal.pone.0155161

**Published:** 2016-05-12

**Authors:** Hafsa Munir, Nguyet-Thin Luu, Lewis S. C. Clarke, Gerard B. Nash, Helen M. McGettrick

**Affiliations:** 1 Institute of Cardiovascular Sciences, College of Medical and Dental Sciences, University of Birmingham, Birmingham, B15 2TT, United Kingdom; 2 Institute of Inflammation and Ageing, College of Medical and Dental Sciences, University of Birmingham, Birmingham, B15 2TT, United Kingdom; French Blood Institute, FRANCE

## Abstract

Mesenchymal stromal cells (MSC) are tissue-resident stromal cells capable of modulating immune responses, including leukocyte recruitment by endothelial cells (EC). However, the comparative potency of MSC from different sources in suppressing recruitment, and the necessity for close contact with endothelium remain uncertain, although these factors have implications for use of MSC in therapy. We thus compared the effects of MSC isolated from bone marrow, Wharton’s jelly, and trabecular bone on neutrophil recruitment to cytokine-stimulated EC, using co-culture models with different degrees of proximity between MSC and EC. All types of MSC suppressed neutrophil adhesion to inflamed endothelium but not neutrophil transmigration, whether directly incorporated into endothelial monolayers or separated from them by thin micropore filters. Further increase in the separation of the two cell types tended to reduce efficacy, although this diminution was least for the bone marrow MSC. Immuno-protective effects of MSC were also diminished with repeated passage; with BMMSC, but not WJMSC, completing losing their suppressive effect by passage 7. Conditioned media from all co-cultures suppressed neutrophil recruitment, and IL-6 was identified as a common bioactive mediator. These results suggest endogenous MSC have a homeostatic role in limiting inflammatory leukocyte infiltration in a range of tissues. Since released soluble mediators might have effects locally or remotely, infusion of MSC into blood or direct injection into target organs might be efficacious, but in either case, cross-talk between EC and MSC appears necessary.

## Introduction

Mesenchymal stromal cells (MSC) are multi-potent tissue-resident precursors which may differentiate for tissue repair but are also able to modulate immune responses in their undifferentiated state [1]. Numerous studies, for instance, have demonstrated the ability of MSC to suppress T-cell proliferation and differentiation of dendritic cells (e.g. reviewed [2–3]). In addition, we have shown recently that cross-talk between MSC and endothelial cells (EC) down-regulated leukocyte recruitment by EC responding to inflammatory cytokines [4]. Thus, MSC may be endogenous regulators of leukocyte entry into tissue, or might be delivered therapeutically to limit acute inflammatory infiltrates or to resolve chronic inflammatory disease.

Several questions arise in relation to these regulatory effects. It is not known whether the ability of MSC to modulate leukocyte recruitment is tissue specific or whether exogenous MSC derived from different sources have equal therapeutic potential in this respect. Tissue specificity is suggested by growing evidence that the MSC niche varies between tissues and that diversity in tissue microenvironment lead to functional differences [5–8]. These variations between MSC may not be maintained after extraction and cell culture, since in general, immunomodulatory effects of MSC are thought to diminish with *in vitro* expansion [9–12]. Nevertheless, MSC from bone marrow (BMMSC) have been reported to inhibit lymphocyte proliferation to a similar [13–14] or lesser extent than those from adipose tissue (ADMSC) [15] or placental-derived MSC [16]. *In vivo*, systemic administration of human umbilical cord-derived or BMMSC ameliorated markers of disease in a murine model of Systemic Lupus Erythematosus [16–17]. On the other hand, murine BMMSC were more effective than ADMSC at preserving tissue viability and promoting angiogenesis in response to hind limb ischemia [18].

It is also uncertain how important for their efficacy are the proximity or contact of MSC with EC, and the route of delivery used for therapy. Most pre-clinical *in vivo* studies have used intravenous infusion of MSC, with evidence on balance showing therapeutic benefit [19]. Since MSC have a very low homing efficiency with few cells reaching the target tissue [20], this suggests that MSC may release soluble mediators systemically that exert effects on distant tissues [21]. However, effects of MSC have also been shown to be promoted by contact with target cells such as leukocytes or EC (reviewed by [2]). The ability of MSC to dampen the inflammatory response of leukocytes is greater when direct contact is made [22–25]. In addition, intra-articular injection of MSC reduced inflammation to a greater extent than intravenous infusion in murine collagen-induced arthritis [26]. One might suggest that site-specific injection of MSC, allowing them to come into close contact with vascular endothelium, would be optimal in therapy. However, experimental evidence is lacking as to how important contact is for MSC-EC interactions that regulate leukocyte recruitment specifically.

Residing in the perivascular niche, MSC have the potential to communicate directly with neighbouring endothelium to regulate leukocyte recruitment during inflammation [4, 27–31]. However, very few studies have examined this. In response to pro-inflammatory cytokines, such as TNFα, EC up-regulate adhesion molecules, chemokines and lipid mediators necessary to support the multi-step leukocyte recruitment cascade. Conditioned media from human BMMSC have been reported to reduce the adhesion of a monocytic cell line (U937) to TNFα-stimulated pulmonary endothelial cells *in vitro*, by tightening endothelial adheren junctions (VE-cadherin and β-catenin) [32]. Indeed, we have previously demonstrated that co-culture of BMMSC in direct contact with endothelial cells suppressed TNFα-induced recruitment of circulating neutrophils and lymphocytes [4].

In this study we compared the potency of MSC isolated from different sources [bone marrow (BM), Wharton's jelly (WJ), and trabecular bone (TB)] in regulating the recruitment of neutrophils to cytokine-treated EC, a key process in inflammation. We hypothesised that MSC from different tissue sources may differ in their ability to suppress endothelial recruitment of neutrophils. To mimic conditions where MSC had different proximities to EC, we utilised distinct models: MSC were incorporated within an endothelial monolayer; MSC and EC were cultured on opposite sides of a Transwell filter or MSC were cultured below but separate from EC cultured on a Transwell filter above. We observed that the different MSC types were all capable of suppressing neutrophil recruitment, although the required proximity varied. Conditioned media from co-cultures were also bioactive, and IL-6 was identifiable as a common soluble mediator of suppression. Our data suggest that the ability of endogenous MSC to limit the inflammatory infiltrate through cross-talk with EC is a characteristic shared by diverse tissues. Isolated MSC may have varying potency and requirement for contact with EC for their therapeutic effects depending on their origin. However, released soluble mediators might have effects on inflammation in a target organ if cross-talk with EC is initiated by infused MSC in a remote organ as well as by locally injected MSC.

## Materials and Methods

### Isolation, culture and characterisation of human MSC

Commercially available primary human bone marrow MSC (BMMSC; Lonza Ltd., Basel, Switzerland) were obtained from healthy donors at passage 2 and expanded three times in culture (i.e., to passage 5) in MSCGM Bulletkit (Lonza Ltd., Basel, Switzerland). Based on manufacturer’s information, cells had undergone ~11–12 doublings at passage 5, and underwent ~2.25 population doublings per passage.

Foetal Wharton’s jelly-derived MSC (WJMSC) were isolated from umbilical cords and expanded to passage 3 as previously described [33]. Briefly, blood vessels were resected and tissue pieces were suspended in phosphate buffered saline (PBS) containing 1mg/ml collagenase type II and 1mg/ml hyaluronidase (all from Sigma-Aldrich, Poole, UK) at 37°C on a rotator for 5h. The cell suspension was filtered and centrifuged at 400g for 5min. WJMSC were resuspended in Low Glucose DMEM with stable L-Glutamine (Biosera, ZI du Bousquet, France) supplemented with 10% foetal calf serum (FCS), 100U/ml penicillin and 100μg/ml streptomycin (all from Sigma). At passage 3 WJMSC had undergone ~8–9 population doublings, and consistently underwent ~2.5 population doublings per passage.

Trabecular bone explants were obtained from elderly osteoarthritis patients (above the age of 60) undergoing joint replacement surgery (in collaboration with Dr Andrew Filer, University of Birmingham, UK). Bone explants were transferred to culture flasks and grown in MSC medium for 2 weeks to allow trabecular bone-derived MSC (TBMSC) to migrate away from the tissue, at which point the fragments were removed. Adherent TBMSC were then cultured to confluence and expanded to passage 3 (~10–11 population doublings). Although BMMSC and TBMSC were isolated from the same tissue source, BMMSC were obtained from BM aspirates while TBMSC were isolated from explant cultures of trabecular bone fragments.

WJMSC and TBMSC were used at passage 3 and BMMSC at passage 5 for experiments, unless otherwise stated.

**Fig 1:**
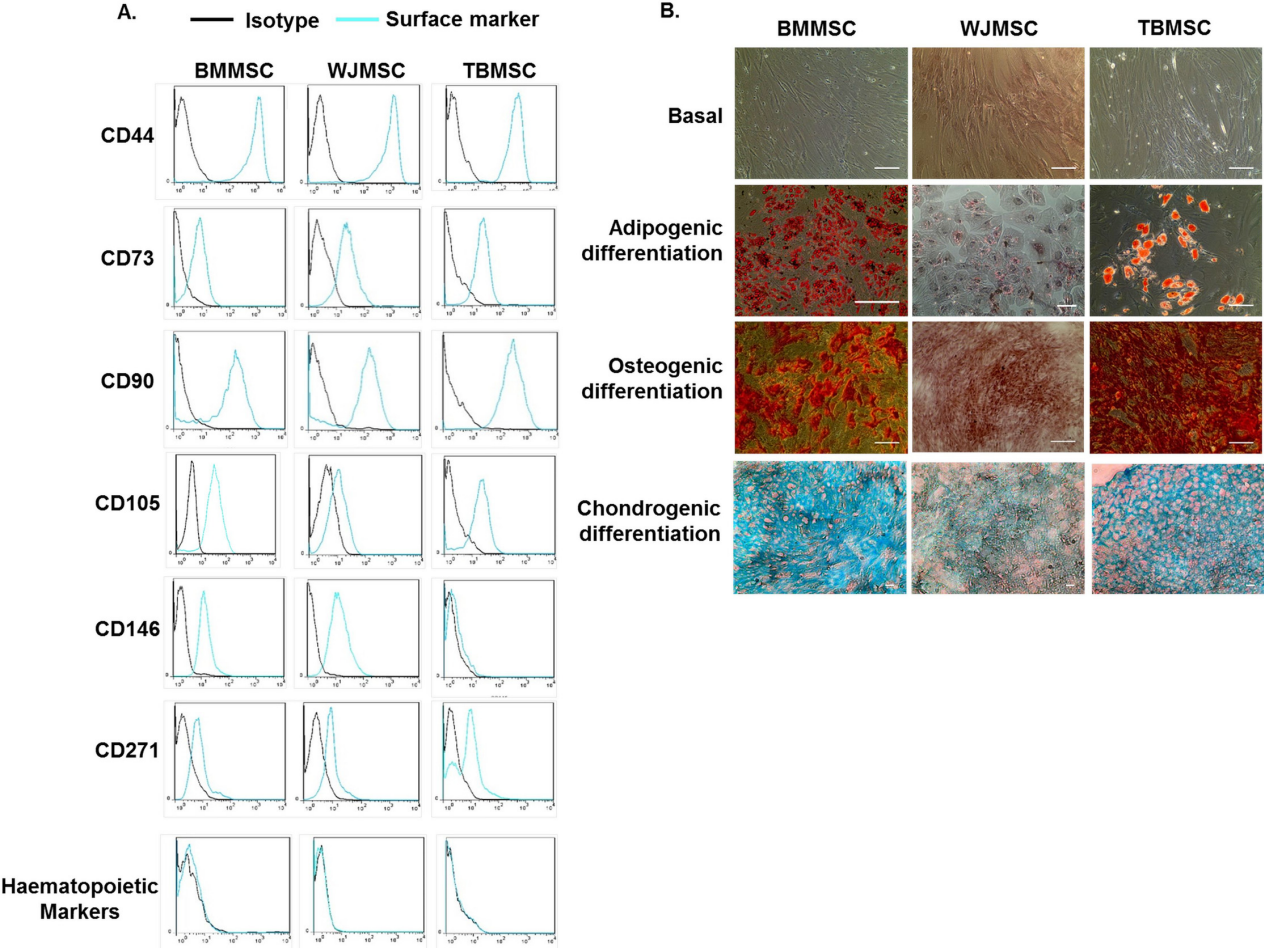
Characterisation of MSC isolated from different tissue sources. (A) Expression of MSC markers CD44, CD73, CD90, CD105, CD146, CD271 and combined expression of the haematopoietic markers CD14, CD20, CD34 and CD45 by BMMSC, WJMSC, or TBMSC by flow cytometry. Data are expressed as representative histograms compared to isotype controls and are representative of n = 3 independent experiments using 3 different MSC donors. (B) BMMSC, WJMSC, and TBMSC were differentiated towards osteoblast, adipocyte, or chrondocyte lineage for 14 or 21 days. Adipogenic, osteogenic and chrondogenic differentiation were assessed using Oil Red O, Alizarin Red or Alcian Blue to stain lipid droplets, calcium deposits or cartilage specific proteoglycans respectively. Phase contrast and colour micrographs are representative of n = 3 independent experiments using 3 different MSC donors. Scale bar represents 10μm.

All cultured cell populations were characterised as MSC based on the International Society for Cell Therapies criteria for defining MSC [34] (Fig 1). For surface marker expression, MSC were incubated for 20min at 4°C with one of the following antibodies diluted in PBS with 0.15% bovine serum albumin (Sigma): CD44-APC, CD73-FITC, CD90-BV421, CD105-PerCP-Cy5.5, CD271-AF647, CD34-PE, CD45-PE, IgG1-FITC, IgG1-PerCP-Cy5.5, IgG2-APC,IgG1-AF647 (all from BD Biosciences), CD146-AF647, IgG1-BV421 (Biolegends; London, UK),CD14-PE, CD20-PE (Immunotools, Friesoythe, Germany), IgG1-PE (ebiosciences). MSC were evaluated using a Cyan ADP flow cytometer and data were analysed offline using Summit 4.3 (both Beckman Coulter Inc., Pasadena, USA). Data are expressed as the percentage of cells positively expressing the marker of interest using the isotype control to gate cells with negative expression (Table 1; Fig 1A).

**Table 1:** Percentage of MSC expressing surface markers.

Surface Marker	BMMSC	WJMSC	TBMSC
CD44	97.94 ± 0.67	98.34 ± 0.56	99.07 ± 0.88
CD73	96.91 ± 1.58	99.47 ± 0.26	94.68 ± 0.90
CD90	97.81 ± 0.69	98.06 ± 1.07	97.52 ± 2.00
CD105	97.61 ± 0.47	96.39 ± 1.46	90.50 ± 4.68
CD146	46.08 ± 6.12	90.71 ± 5.10	24.64 ± 9.46
CD271	32.79 ± 0.50	53.91 ± 3.23	19.31 ± 8.61
Haematopoietic markers	9.31 ± 6.17	3.98 ± 1.86	6.47 ± 0.45

Haematopoietic markers, CD14, CD20, CD34 and CD45, were stained in combination in a single tube. Data are percentage mean ± SEM, for n = 5 independent experiments, with the exception of staining for CD146 and CD271 on all MSC types and all analysis of TBMSC where n = 3.

To assess differentiation of MSC, cells were cultured for 14 or 21 days in Osteogenic Differentiation, Adipogenic Induction (both from Lonza) or MesenCult-ACF Chondrogenic Differentiation (STEMCELL Technologies, Cambridge, UK) according to the manufacturer’s instructions. All samples were fixed in 10% neutral buffered formalin (Sigma) for 30min. MSC-derived adipocytes were treated with 60% isopropanol (Sigma) and stained for 30min with 0.3% Oil Red O (Sigma) dissolved in isopropanol. MSC-derived osteoblasts were stained for 45min with Alizarin Red dye (Sigma) dissolved in distilled water. Samples were washed in distilled water and counterstained with haemotoxylin solution (Sigma). MSC-derived chrondocytes were dehydrated in ethanol, embedded in paraffin, sectioned and stained for 24h with 10μg/ml Alcian Blue 8GX (Alfa Aesar, Lancashire, UK) dissolved in a 3:2 ethanol to acetic acid solution and nuclear fast red (Vector, Peterborough, UK). Cells were imaged using phase contrast microscopy and digitised images were acquired using a EVOS FL Imaging System (Thermo Scientific, Loughborough, UK) (Fig 1B).

### Isolation and culture of EC

Human umbilical vein endothelial cells (HUVEC) were isolated from umbilical cords as previously described [33, 35] and cultured in Medium 199 (Life Technologies, Paisley, UK) supplemented with 20% FCS, 35μg/ml Gentamycin, 10ng/ml epidermal growth factor, 1μg/ml hydrocortisone (all from Sigma) and 2.5 μg/ml Amphotericin B (Life Technologies).

### Isolation of neutrophils

Venous blood was collected from healthy donors into EDTA tubes (Sarstedt, Leicester, UK). Neutrophils were isolated by two-step histopaque density centrifugation as previously described [4, 33]. Purified neutrophils were washed twice in PBS containing 1mM Ca^2+^, 0.5mM Mg^2+^ and 0.15% bovine serum albumin (PBSA; all from Sigma) at 250g for 5min. Neutrophils were counted and re-suspended to 2x10^6^ cells/ml in PBSA.

### Ethics

The study was conducted in compliance with the Declaration of Helsinki. All human samples were obtained with written, informed consent and approval from the Human Biomaterial Resource Centre (Birmingham, UK), West Midlands and Black Country Research Ethics Committee or University of Birmingham Local Ethical Review Committee.

### EC and MSC co-culture in channel slides

**Fig 2:**
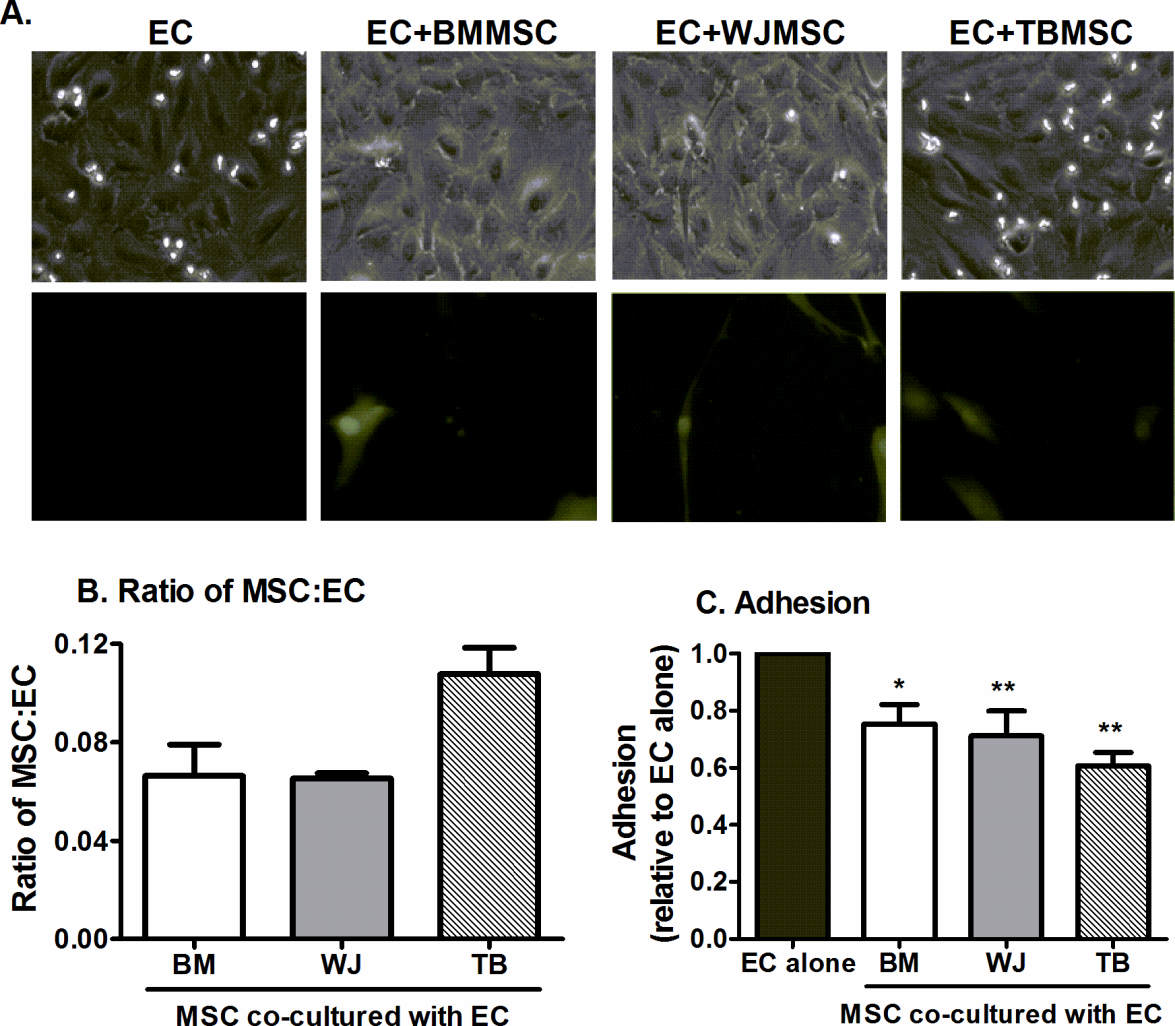
Effect of MSC from different tissues on neutrophil recruitment when cultured in direct contact with EC. BMMSC, WJMSC, or TBMSC were cultured in direct contact with EC in channel slides for 24h before addition of 100U/ml TNFα for 4h. (A) MSC labelled with Cell Tracker Green integrated into the EC monolayer. Fluorescent micrographs are representative of n = 3 independent experiments using 3 different MSC donors. (B) Ratio of MSC to EC for each co-culture assessed after the adhesion assay. ANOVA showed a significant effect of MSC source on MSC:EC ratio, p<0.05; n = 3 independent experiments, using 3 different MSC donors for each MSC type. (C) Neutrophil adhesion expressed as a proportion of that observed on the paired EC mono-culture control. ANOVA showed a significant effect of culture conditions on adhesion, p<0.01; n = 5 independent experiments, using 3 different BMMSC, 5 WJMSC and 5 TBMSC donors. Data are mean ± SEM, incorporating a different EC and neutrophil donor in each experiment. * = p<0.05 and ** = p<0.01 compared to EC mono-cultures a by Dunnett post-test.

Primary EC were dissociated using trypsin/EDTA (Sigma) and seeded in pre-coated channel slides (μ-Slide VI; ibidi GmbH, Martinsried, Germany) at a density that would yield a confluent monolayer within 24h [4, 33]. After 24h, MSC were dissociated, counted and labelled with 5μM Cell Tracker Green (Life Technologies) for 30min (1.5x10^5^ cells/ml in MSCGM), and seeded onto the EC (~13,500 MSC per channel) and allowed to settle for 1h as described [4, 33]. Non-adherent cells were removed by washing with MSCGM, and the numbers of EC and of fluorescent MSC were counted in 5 fields (Fig 2A). The ratio of MSC:EC was then calculated. Cells were co-cultured in direct contact for 24h prior to treatment with or without 100U/ml tumour necrosis factor–alpha (TNFα; R&D Systems, Abingdon, UK) for a further 4h. EC mono-cultures were set up in parallel as controls.

### EC and MSC co-culture on Transwell filters

MSC-EC co-cultures were established on opposite sides of 0.4μm pore Transwell filter inserts (BD Pharmingen, Cowley, UK) as previously described [4, 33]. Briefly, MSC (5x10^5^) were seeded onto inverted filters and cultured for 24h. EC were then seeded on the inner surface of the inserts in close proximity. Alternatively, co-cultures were established by seeding MSC onto the bottom of a 6-well plate for 24h and seeding EC above inside the filter so the two cell types were separated [36]. Cells were co-cultured for 24h prior to treatment with TNFα as above. Parallel endothelial mono-cultures were set up as controls.

To investigate the bioactivity of co-culture conditioned media, supernatants were collected from EC and MSC mono- and co-cultures at 24h. Fresh EC mono-cultures were treated with conditioned media for 24h prior to stimulation with TNFα in the same conditioned media for a further 4h.

In some experiments, a neutralising antibody against either IL-6 (5μg/ml; clone 6708) or a function blocking antibody against membrane IL-6 receptor (IL-6R; 5μg/ml; clone 17506; all from R&D Systems) was added when co-cultures were established in close proximity on opposite sides of porous insert and were present throughout the co-culture and cytokine-stimulation.

### Flow-based adhesion assay

Flow based adhesion assays were performed for channel slides or filters incorporated into a custom-made parallel-plate flow chamber using phase-contrast digital microscopy as previously described [4, 33]. Purified leukocytes were perfused over EC for 4min followed by washout with cell-free PBSA at 0.05PA (microslide model) or 0.1Pa (filter model). Digitised recordings of 5–10 random fields were made at 2 and 9 minutes after the end of the neutrophil bolus to assess neutrophil adhesion and transmigration respectively. Images were analysed offline using Image-Pro Plus software (Media Cybernetics, Marlow, UK). Total leukocytes bound to the endothelium were counted, and classified as either: (i) phase bright, rolling, or stationary adherent on the apical surface or (ii) phase-dark, spread and transmigrated under the endothelial monolayer. The numbers of adherent and of transmigrated neutrophils were averaged per field. Values for co-cultures were expressed as a proportion of those observed on the paired EC mono-culture control.

### Quantification of IL-6

Culture supernatants were obtained from unstimulated EC and MSC cultured alone or in co-culture for 24h. IL-6 was quantified using IL-6 DuoSet ELISA (R&D Systems) according to manufacturer's instructions.

### Gene expression analysis

Endothelial mRNA was isolated from EC mono-cultures and EC-BMMSC co-cultures using the RNeasy Mini Kit (Qiagen, Crawley, UK), converted to cDNA and analysed by qPCR using Universal PCR mastermix (Life Technologies) according to manufacturer’s instructions. Primers were bought as Assay on Demand kits from Applied Biosystems. Genes of interest were amplified using the 7900HT Real-Time PCR machine, analysed using SDS 2.2 (Applied Biosystems) and expressed as 2^-ΔCT^ (relative to 18S).

### Statistical analysis

Data are expressed as mean ± SEM, where a different EC and leukocyte donor were used for each experiment. Between 3–5 different MSC donors were incorporated in each experiment for each biological replicate for TB and WJ (i.e. 3–5 biological replicates per experiment), and 2–3 donors for BM. Multi-variant data when paired were analysed using analysis of variance (ANOVA), followed by Bonferroni or Dunnett post-hoc test. Paired t-test was used when multiple parameters were grouped together even though not all conditions were tested in every experiment, but EC mono-culture controls were performed on each occasion. p<0.05 was considered statistically significant.

## Results

### Characterisation of primary MSC isolated from different tissue sources

BMMSC, WJMSC and TBMSC were initially characterised based on the expression of known MSC markers and the capacity to undergo differentiation into adipocytes and osteoblasts [34]. BMMSC, WJMSC, and TBMSC all expressed the markers CD44, CD73, CD90, and CD105 upon their surface, with little if any contaminating haematopoietic progenitors based on the expression of CD14, CD20, CD34, and CD45 (Fig 1A). Interestingly, only BMMSC and WJMSC expressed CD146, while all 3 MSC populations expressed CD271 (Fig 1A). All MSC types exhibited the capacity to differentiate down the adipogenic, osteogenic and chondrogenic lineages, although WJMSC-derived adipocytes were observed to have fewer lipid droplets when compared to BMMSC- or TBMSC-derived adipocytes (Fig 1B).

### Effects of MSC from different tissue sources on neutrophil recruitment by inflamed EC

We previously reported that BMMSC were able to suppress leukocyte recruitment to tumour necrosis factor–alpha (TNFα)-stimulated EC when integrated into the EC monolayer, using channel slides to mimic systemic infusion of MSC [4]. Using this model, we compared the ability of human MSC isolated from different tissues (BM, WJ and TB) to regulate neutrophil recruitment. Although MSC were seeded at the same density, more TBMSC adhered to the EC monolayer than BMMSC and WJMSC (Fig 2A and 2B). Co-culture with MSC from different tissues suppressed neutrophil recruitment to TNFα-stimulated EC to a similar extent when incorporated into the EC monolayer (Fig 2C).

**Fig 3:**
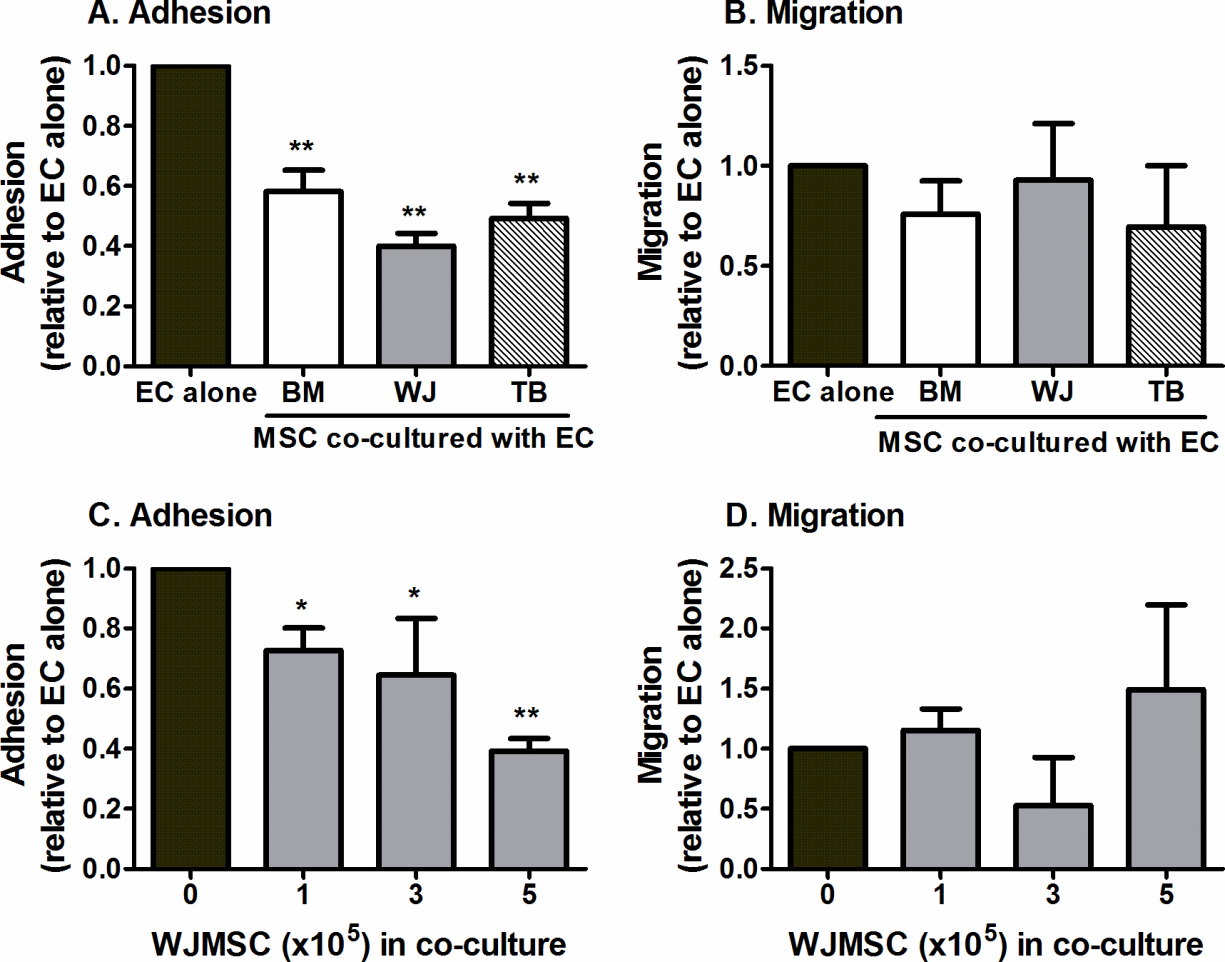
Effect of MSC from different tissues on neutrophil recruitment when cultured on the opposite side of the filter to EC. MSC and EC were cultured on opposite sides of 0.4μm porous filters for 24h prior to stimulation with 100U/ml TNFα for 4h. Co-cultures formed with (A-B) MSC from different sources (BM, WJ, TB) or (C-D) with different numbers of WJMSC. Neutrophil (A,C) adhesion and (B,D) migration were expressed as a proportion of values observed on the paired EC mono-culture control. In A and C, ANOVA showed a significant effect of culture conditions on neutrophil adhesion, p<0.001. Data are mean ± SEM from (A-B) n = 11–13 and (C-D) n = 3–5 experiments incorporating a different EC and neutrophil donor in each. (A-B) 3 different BMMSC, 5 different WJMSC and 5 different TBMSC donors were used and (C-D) 5 different WJMSC donors were used, with the exception of 3x10^5^ cells where 3 different donors were used * = p<0.05 and ** = p<0.01 compared to EC mono-cultures by Dunnett post-test.

To mimic the effect of tissue-resident MSC, or those administered directly into tissue, we cultured MSC and EC in close proximity, but not in direct contact, prior to assessing neutrophil recruitment from flow [4, 30, 33]. Once again, we observed that all 3 MSC types were capable of suppressing neutrophil adhesion to TNFα-stimulated EC to a similar extent (Fig 3A). Co-culture did not have a significant effect on neutrophil migration through the endothelial monolayer when compared to EC cultured alone (Fig 3B). Using WJMSC as an example, we observed that the reduction in neutrophil adhesion increased with increasing number of MSC added to the co-culture, with the greatest effects obtained with 5x10^5^ WJMSC (Fig 3C). The number of neutrophils undergoing transmigration was not significantly altered by the number of MSC (Fig 3D). Further experiments were performed using the filter-based model incorporating 5x10^5^ MSC, unless otherwise stated.

### Effects of passage on immunomodulation by MSC

**Fig 4:**
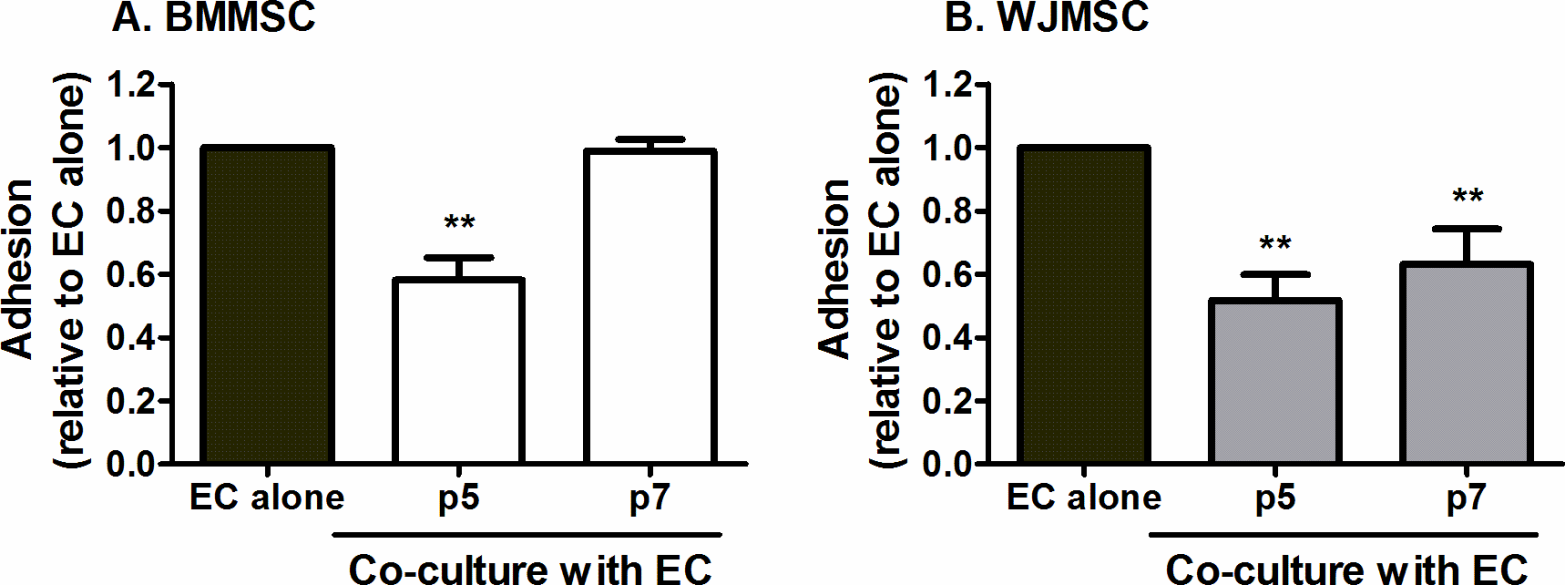
Effects of passage on the ability of MSC to suppress neutrophil recruitment. (A) BMMSC or (B) WJMSC at different passage number were co-cultured with EC on opposite sides of a porous filter for 24h prior to stimulation with TNFα for 4h. Neutrophil adhesion was expressed as a proportion of that observed on the paired EC mono-culture control. In A and B, ANOVA showed a significant effect of passage on neutrophil adhesion, p<0.01. Data are mean ± SEM from (A) n = 13 for EC, n = 13 p5 MSC-EC and n = 3 p7 MSC-EC co-cultures and (B) n = 3 for all data independent experiments using a different EC and neutrophil donor in each experiment. (A) 5 or 3 different BMMSC donors were used at p5 or p7 respectively; and (B) 3 different WJMSC donors were used at both passages. ** = p<0.01 compared to EC mono-cultures by Dunnett post-test.

We tested whether passaging MSC altered their ability to regulate neutrophil recruitment. *Ex vivo* expansion of BMMSC to p7 (Fig 4A) and p9 (data not shown) completely abrogated their ability to suppress neutrophil adhesion, as compared to p5 BMMSC. In contrast, WJMSC maintained the capacity to limit neutrophil recruitment up to p7, compared to p5 WJMSC (Fig 4B) and p3 (data not shown), although the potency of this effect gradually reduced over passage. Effects of passage were not assessed for TBMSC as they grew considerably slower than the other MSC types, presumably due to the fact that the cells were isolated from elderly patients with osteoarthritis.

### Effects of proximity between MSC and EC and of co-culture supernatants on neutrophil recruitment

**Fig 5:**
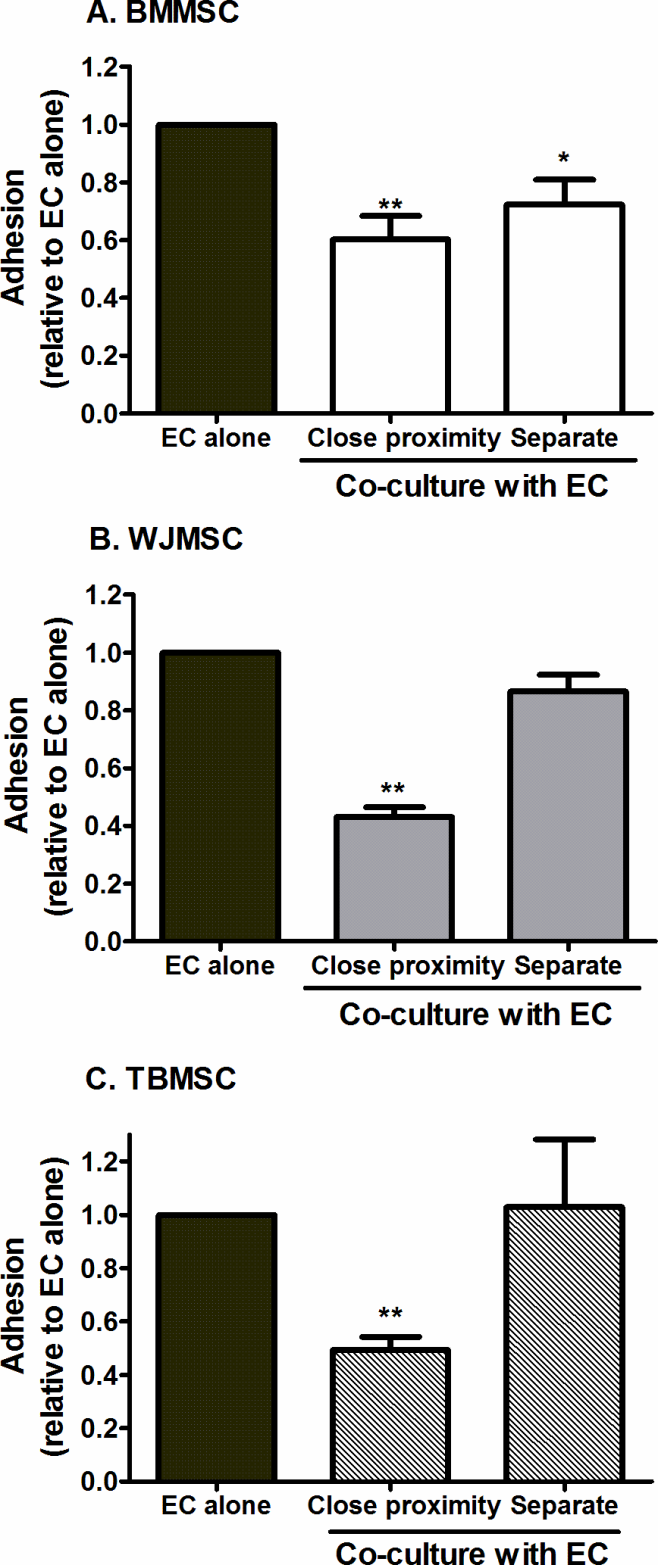
Effects of varying proximity between MSC and EC on neutrophil recruitment. Co-cultures were formed by seeding BMMSC, WJMSC, or TBMSC on the opposite side of a Transwell filter to EC (Close proximity) or by seeding MSC on the plate below EC cultured on a filter (Separate). Neutrophil adhesion was expressed as a proportion of that observed on the paired EC mono-culture control where a different EC and neutrophil donor was used in each experiment. ANOVA showed a significant effect of culture conditions on neutrophil adhesion for each type of MSC, p<0.01. Data are mean ± SEM, n = 11 for EC alone and close proximity co-cultures or n = 3 for separate co-cultures. (A) 5 or 2 different BMMSC donors were used for close proximity or separate cultures respectively, (B) 3 different WJMSC and (C) 3 different TBMSC donors were used for both culture models. * = p<0.05 and ** = p<0.01 compared to EC mono-cultures by Dunnett post-test.

To assess whether close proximity was essential for crosstalk between MSC and EC, MSC were cultured on the plate below, separated by 0.9mm from the EC cultured above on the filter. When MSC and EC were separated, BMMSC were still capable of significantly reducing neutrophil adhesion, albeit to a slightly lesser extent than when the two cell types were cultured on opposite sides of a filter (Fig 5A). In contrast, separating WJMSC or TBMSC from EC during co-culture nullified their inhibitory effects on neutrophil adhesion (Fig 5B and 5C). Thus MSC varied depending on their source in their requirement for proximity to EC for functional efficacy.

**Fig 6:**
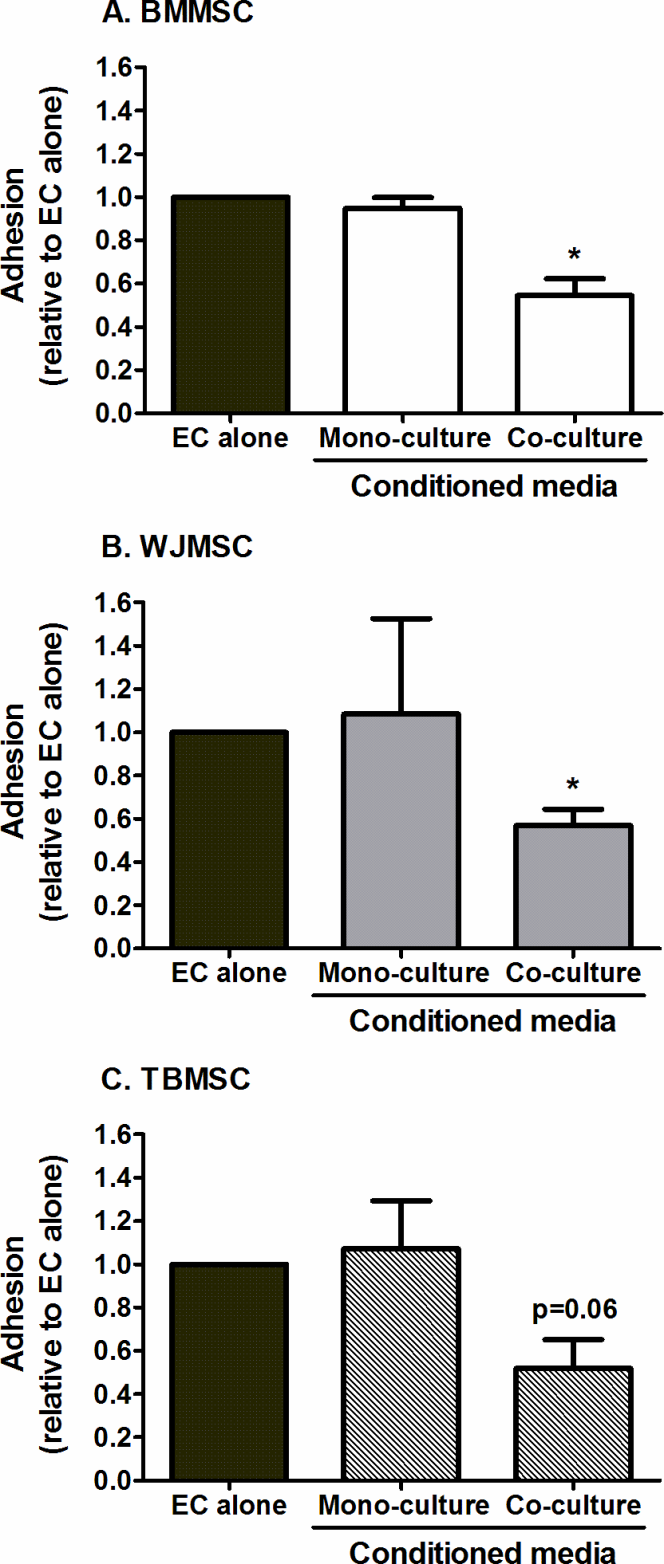
Effects of media conditioned by MSC alone or in co-culture with EC on neutrophil recruitment. EC mono-cultures were treated with conditioned media from BMMSC, WJMSC, or TBMSC either cultured alone or co-cultured with EC on opposite sides of filters for 24h. Not all conditions were performed in all experiments, but treated cells were always compared to paired untreated EC controls. Neutrophil adhesion was assessed at 2min post-perfusion and expressed as a proportion of that observed on the paired EC mono-culture control. ANOVA showed a significant effect of culture conditions on neutrophil adhesion for each type of MSC, p<0.01. Data are mean ± SEM from n = 3 independent experiments using a different EC and neutrophil donor in each experiment. (A) 2 different BMMSC, (B) 3 different WJMSC and (C) 3 different TBMSC donors were used. * = p<0.05 compared to EC cultured without conditioned media by Dunnett post-test.

Next we examined whether MSC-EC co-culture on opposite sides of filters led to the release of bioactive soluble agents, as previously described for MSC integrated into EC monolayers [4]. EC mono-cultures were incubated for 24h with conditioned media from MSC mono-cultures or co-cultures prior to cytokine-stimulation. Co-culture conditioned media from each MSC type mimicked the effect of MSC-EC co-culture, inhibiting neutrophil adhesion to TNFα-treated EC mono-cultures (Fig 6). In contrast, media from MSC mono-culture had no effect on adhesion (Fig 6). Thus all MSC types caused the release of bioactive immuno-protective agents when cultured with EC, which regulated the inflammatory infiltrate.

### Role of IL-6 in regulating recruitment in MSC co-cultures

**Fig 7:**
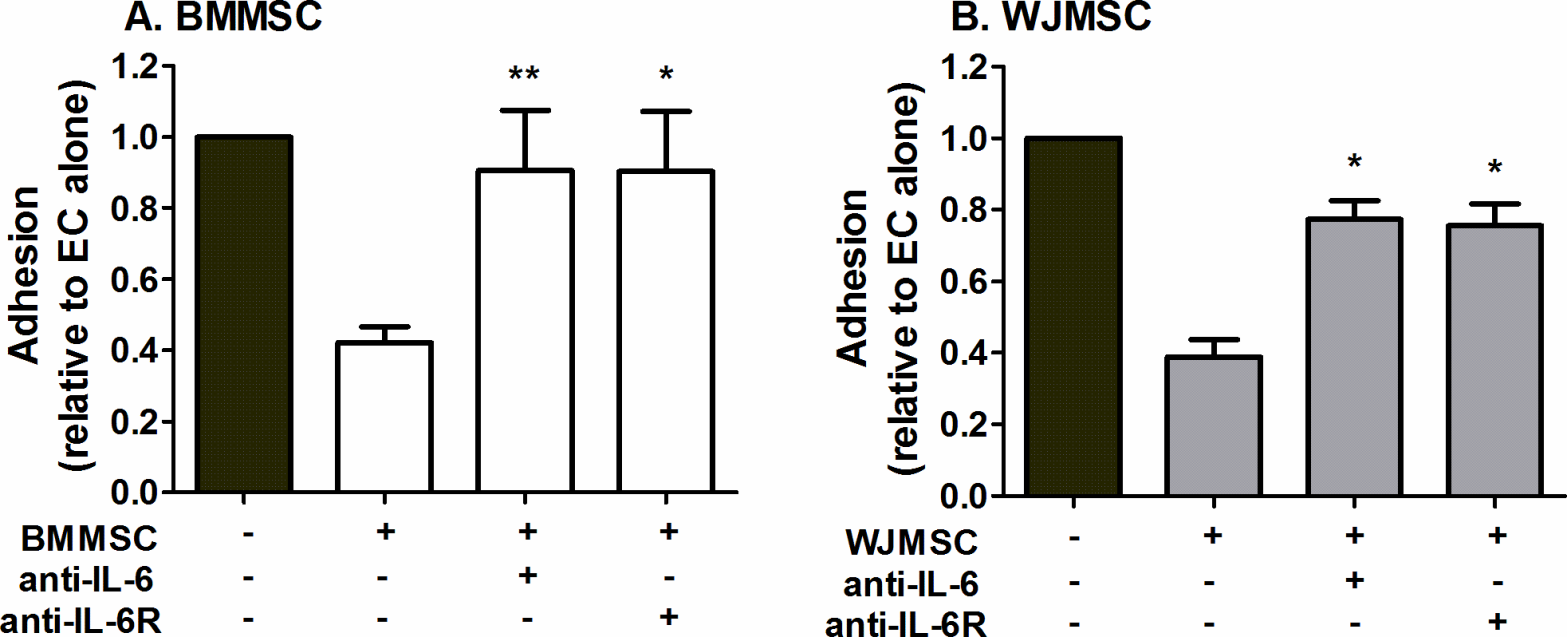
Effect of blocking the actions of IL-6 on the immunosuppressive effects of MSC in co-culture. (A) BMMSC or (B) WJMSC co-cultures were cultured in close proximity, on opposite sides of a porous filters for 24h and then treated with neutralising antibodies against IL-6 or a function blocking antibody against IL-6R for the duration of the co-culture and cytokine treatment. Neutrophil adhesion was expressed as a proportion of that observed on the paired EC mono-culture. ANOVA showed a significant effect of co-culture treatment on neutrophil adhesion in (A) (p<0.05) and (B) (p<0.01). Data are mean ± SEM, n = 3–4 independent experiments using a different EC and neutrophil donor in each experiment. (A) 3 different BMMSC and (B) 4 different WJMSC donors were used. * = p<0.05 and ** = p<0.01 compared to untreated MSC co-cultures by Dunnett post-test.

We previously showed that IL-6 contributed to the reduction in leukocyte adhesion when EC were cultured in direct contact with BMMSC or in close proximity to fibroblasts [4, 30]. Here inhibition of IL-6 signalling using antibodies that targeted soluble IL-6 or membrane IL-6R both significantly reduced the inhibitory effects of BMMSC and WJMSC in co-culture (Fig 7).

### IL-6 secretion by co-cultures

**Fig 8:**
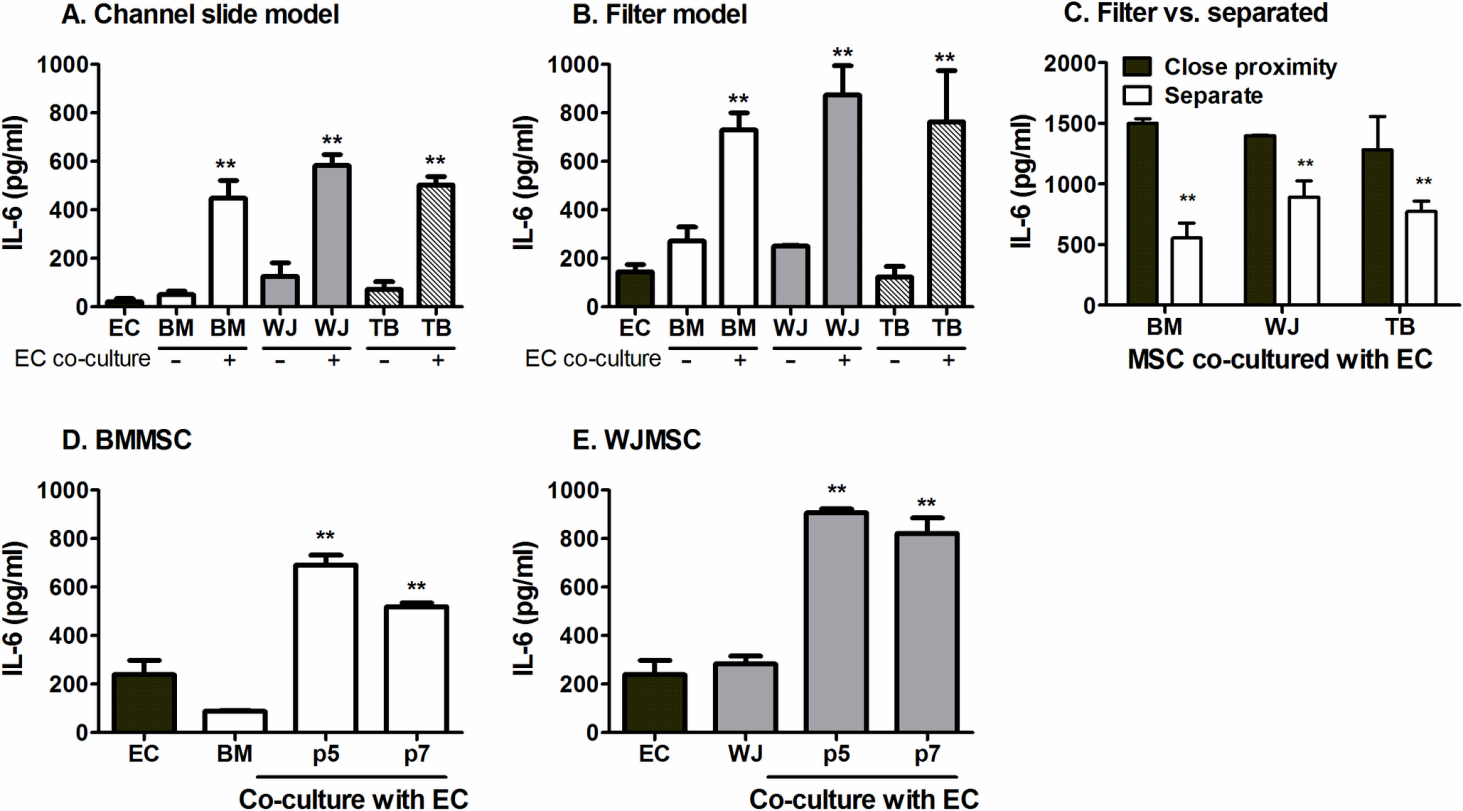
Effects of co-culture, of proximity between MSC and EC and MSC passage on IL-6 secretion. MSC-EC co-cultures were formed (A) in direct contact using channel slides; (B, D-E) in close proximity on opposite sides of a porous filter; or (C) in close proximity or with MSC seeded below and separate from EC on the filter above. EC and MSC mono-cultures (at p3 for WJMSC and p5 for BMMSC) were set up as controls. IL-6 release into supernatants was assessed after 24h. In (D) and (E), ANOVA showed a significant effect of culture conditions, p<0.01. Data are mean ± SEM from (A) n = 4–8 (B) n = 3–19 where EC mono-cultures (n = 19); BMMSC mono-cultures (n = 13); EC:BMMSC (n = 16); WJMSC mono-cultures (n = 3); EC:WJMSC (n = 10); TBMSC mono-cultures (n = 4); EC:TBMSC (n = 7), (C-E) n = 3 independent experiments using a different EC and neutrophil donor in each experiment. (A-E) 5 different BMMSC, 3 different WJMSC and 3 different TBMSC donors were used. ** = p<0.01 compared to the sum of the EC and respective MSC mono-cultures supernatant unless otherwise indicated by paired t-test in A-C, or by Dunnett post-test in D-E.

Additionally, we have reported that BMMSC co-culture with EC induced a marked increase in IL-6 secretion when the two cell types were in direct contact [4]. Here, we detected a significantly more IL-6 released from co-cultures incorporating all MSC types, either incorporated within the EC monolayer (Fig 8A) or in close proximity on opposite sides of a filter (Fig 8B), compared to the sum of the EC and MSC mono-cultures. Separating MSC from EC reduced the amount of IL-6 secreted in comparison to when the two cell types were cultured on opposite sides of a filter (Fig 8C). However, the level of IL-6 produced when MSC-EC were separated was still higher than that released from individual mono-cultures (Fig 8C). Similar observations were made for MSC from all sources, suggesting IL-6 was a common soluble effector.

We also measured IL-6 generated by co-cultures incorporating BMMSC or WJMSC at a higher passage to evaluate whether IL-6 levels correlated with changes in the suppression of neutrophil recruitment upon MSC passage shown in Fig 4. Perhaps surprisingly, IL-6 secretion by co-cultures was not affected by passage and remained much higher than mono-cultures for both BMMSC and WJMSC (Fig 8D and 8E). Thus although IL-6 was a common effector of immunomodulation, levels were not closely associated with the degree of immunosuppression during passage and indicated a role for additional soluble co-factor(s) that presumably did vary with passage.

### Analysis of gene expression in endothelial cells upon co-culture with BMMSC

To further investigate the mechanism underlying the suppression of neutrophil adhesion, we analysed the expression of adhesion molecules by EC using qPCR. BMMSC tended to reduce the gene expression of the capture receptor E-selectin (0.07 ± 0.08 ^2-ΔCT^) and the β_2_-integrin ligand ICAM-1 (0.84 ± 0.18 x10^-^6 ^2-ΔCT^) by EC in co-cultures compared to EC mono-cultures (19.04 ± 7.6 ^2-ΔCT^ and 27 ± 9.26 x10^-^6 ^2-ΔCT^ respectively; mean ± SEM, n = 3).

## Discussion

MSC are tissue-resident stromal cells with immunomodulatory and reparative properties, which we recently showed to be able to suppress leukocyte recruitment [4]. Here we compared the ability of MSC isolated from bone marrow aspirates, umbilical cord Wharton's jelly and trabecular bone explants to regulate adhesion of flowing neutrophils to cytokine-treated EC. Co-culture models were used with different degrees of proximity between the MSC and EC. MSC from these tissues showed similar capability in suppressing neutrophil recruitment. However, WJMSC appeared to retain their effects at higher passage than BMMSC, and we were not able to maintain TBMSC in culture to high passage. On the other hand, BMMSC retained a stronger effect when physically separated from EC by a small gap (~900μm) than the other MSC types. Conditioned media from all co-cultures suppressed neutrophil recruitment, and IL-6 was identified as a common bioactive mediator. Immuno-protective effects diminished with passage, although IL-6 levels remained high indicating that IL-6 did not act alone. Thus, MSC may be tissue-resident cells that communicate with EC, acting as endogenous regulators of inflammatory infiltrates. From a therapeutic standpoint, potency, requirement for close contact with EC and variation with passage varied slightly for MSC from the different tissues.

In general, MSC mediate their effects through the release of soluble factors, although these effects can be enhanced by direct MSC-target cell interaction (reviewed by [2]). The immuno-protective effects we report here were transferable in the conditioned media from all MSC types in co-culture. Importantly, we observed that a two-way conversation between MSC and EC was essential to generate the bioactive agent(s), as conditioned media from MSC mono-cultures were unable to suppress neutrophil recruitment. The need for cross-talk for effects on recruitment of leukocytes is consistent with previous reports [4, 32, 33, 37]. Specifically, we found that cross-talk was necessary to generate the high levels of IL-6 found in co-culture supernatants, which originated mainly from the MSC [4]. Here, IL-6 was an active agent in all the conditioned media. IL-6 can have either pro- or anti-inflammatory effects in different conditions, including increasing or decreasing leukocyte recruitment [4, 29, 30], or switching recruitment from neutrophils to mononuclear leukocytes (e.g. [4, 29, 30, 38]), presumably depending on other co-factors present. We recently showed complementary roles for IL-6, soluble IL-6 receptor and TGFβ in immunosuppression when MSC and EC were cultured in direct contact [4]. Here although IL-6 was a common effector of immunomodulation, levels remained high during passage as the degree of immunosuppression decreased; indicating role(s) for additional soluble co-factor(s) that presumably did vary with passage. Moreover, the partial reduction in gene expression for key adhesion molecules (E-selectin and ICAM-1), may also help to explain, in part at least, changes in recruitment observed.

Given the clear role for released soluble mediators, we wondered whether cell-cell contact was required for MSC-EC cross-talk. Culturing MSC and EC on opposite sides of thin microporous filters separated the 2 cell types by ~10μm, although the 0.4μm pores may have allowed interactions via the tips of protruding pseudopods. This format was as effective as mixed culture on a surface [4]. Increasing the distance between MSC and EC tended to reduce efficacy of immuno-suppression. Efficacy was retained best by BMMSC, which may indicate a variation in the sensitivity of the MSC from different tissues to substance(s) released by EC. The distance from the filter to the bottom of the well in the separated co-cultures was ~900μm, which is larger than the expected distance *in vivo* between tissue-resident cells and local blood microvessels ~200um. Thus *in vivo* MSC may be able to communicate with EC with or without direct contact to modulate inflammatory infiltrates. These considerations are relevant to therapeutic utility of MSC. Delivery via intravascular infusion would bring MSC directly to the endothelial surface. While the MSC might be delivered to a number of sites as well as the target organ, soluble mediators generated by cross-talk at those sites could also have efficacy via release into the systemic circulation (e.g. [21]). Our results predict that direct injection of MSC into tissue would also be efficacious, if the cells achieved adequately close contact with the vascular endothelium. It is also worth considering that knowledge of the bioactive agents generated in co-cultures along with IL-6 might allow their delivery for 'cell-free' therapy, which mimics the protective effects of MSC.

Previous studies showed, as here, that the immunomodulatory effects of MSC varied with the time in culture or passaging [9–12] and ratio of MSC-to target cell [39–41]. The effects of passage could have significant impact on clinical benefit. Here we demonstrated that WJMSC better maintained their potency in culture with passage, compared to BMMSC whose effects on EC were lost by passage seven. Others found that MSC derived from umbilical cord (effectively WJMSC) retained their effects on T-cell proliferation to much higher passage [9, 12]. Comparison of the potency of different types of MSC is difficult because it may depend on readout chosen. For instance, MSC derived from umbilical cord had similar effects on mononuclear cell proliferation compared to BMMSC [21]. We found that while BMMSC and WJMSC induced similar suppression of neutrophil recruitment, BMMSC lost this capacity more quickly with *in vitro* expansion, but could operate at greater distances from EC in comparison to WJMSC. TBMSC were also able to suppress recruitment but proved difficult to expand beyond passage three.

In conclusion, endogenous MSC from several tissues were able to communicate with EC to limit inflammatory infiltrates, producing a common bioactive agent in IL-6. There was no clearly-superior type of MSC for therapeutic use, although the stability in culture of WJMSC may be advantageous. Our results support the concept that MSC are endogenous tissue-resident regulators of inflammation. Variations in the MSC niche between organs and the capacity of MSC to respond to changes in the local microenvironment [7–8, 42–44] suggest that modulation of their generic immunomodulatory capability might contribute to tissue-specific variations in susceptibility to inflammation or patterns of leukocyte recruitment. Mesenchymal stromal cells from a variety of healthy tissues also exhibit immunosuppressive capabilities [4, 28, 30], while cells from diseased tissue may induce inflammatory infiltrates [29–30]. The foregoing suggests the intriguing possibility that changes in the local MSC microenvironment could adversely influence MSC function.

## References

[1] Pal R, Hanwate M, Jan M, Totey S. (2009) Phenotypic and functional comparison of optimum culture conditions for upscaling of bone marrow-derived mesenchymal stem cells. J. Tissue Eng. Regen. Med. 3, 163–174. 10.1002/term.143 19229888

[2] Munir H, McGettrick HM. (2015) Mesenchymal stem cells therapy for autoimmune disease: risks and rewards. Stem Cells Dev. 24, 2091–2100. 10.1089/scd.2015.0008 26068030

[3] Le Blanc K, Mougiakakos D. (2012) Multipotent mesenchymal stromal cells and the innate immune system. Nat. Rev. Immunol. 12, 383–396. 10.1038/nri3209 22531326

[4] Luu NT, McGettrick HM, Buckley CD, Newsome PN, Rainger GE, Frampton J, et al (2013) Crosstalk between mesenchymal stem cells and endothelial cells leads to downregulation of cytokine-induced leukocyte recruitment. Stem Cells 31, 2690–2702. 10.1002/stem.1511 23939932

[5] Magatti M, De Munari S, Vertua E, Gibelli L, Wengler GS, Parolini O (2008) Human amnion mesenchyme harbors cells with allogeneic T-cell suppression and stimulation capabilities. Stem Cells 26, 182–192. 17901399 10.1634/stemcells.2007-0491

[6] Chang C-J, Yen M-L, Chen Y-C, Chien C-C, Huang H-I, Bai C-H, et al (2006) Placenta-derived multipotent cells exhibit immunosuppressive properties that are enhanced in the presence of interferon-gamma. Stem Cells 24, 2466–2477. 17071860 10.1634/stemcells.2006-0071

[7] Chen P-M, Yen M-L, Liu K-J, Sytwu H-K, Yen B-L (2011) Immunomodulatory properties of human adult and fetal multipotent mesenchymal stem cells. J. Biomed. Sci. 18, 49 10.1186/1423-0127-18-49 21762539 PMC3156728

[8] Wolbank S, Peterbauer A, Fahrner M, Hennerbichler S, van Griensven M, Stadler G, et al (2007) Dose-dependent immunomodulatory effect of human stem cells from amniotic membrane: a comparison with human mesenchymal stem cells from adipose tissue. Tissue Eng. 13, 1173–1183. 17518752 10.1089/ten.2006.0313

[9] Yu K-R, Lee JY, Kim H-S, Hong I-S, Choi SW, Seo Y, et al (2014) A p38 MAPK-Mediated Alteration of COX-2/PGE2 Regulates Immunomodulatory Properties in Human Mesenchymal Stem Cell Aging. PLoS One 9, e102426 10.1371/journal.pone.0102426 25090227 PMC4121064

[10] Zhuang Y, Li D, Fu J, Shi Q, Lu Y, Ju X (2014) Comparison of biological properties of umbilical cord-derived mesenchymal stem cells from early and late passages: Immunomodulatory ability is enhanced in aged cells. Mol. Med. Rep. 11, 166–174. 10.3892/mmr.2014.2755 25339265 PMC4237101

[11] Li XY, Ding J, Zheng ZH, Wu ZB, Zhu P (2012) Long-term culture in vitro impairs the immunosuppressive activity of mesenchymal stem cells on T cells. Mol Med Rep 6, 1183–1189. 10.3892/mmr.2012.1039 22923041

[12] Giuliani M, Fleury M, Vernochet A, Ketroussi F, Clay D, Azzarone B, et al (2011) Long-lasting inhibitory effects of fetal liver mesenchymal stem cells on T-lymphocyte proliferation. PLoS One 6, e19988 10.1371/journal.pone.0019988 21625521 PMC3098287

[13] Puissant B, Barreau C, Bourin P, Clavel C, Corre J, Bousquet C, et al (2005) Immunomodulatory effect of human adipose tissue-derived adult stem cells: Comparison with bone marrow mesenchymal stem cells. Br. J. Haematol. 129, 118–129. 15801964 10.1111/j.1365-2141.2005.05409.x

[14] Roemeling-van Rhijn M, Khairoun M, Korevaar SS, Lievers E, Leuning DG, Ljzermans JN, et al (2013) Human Bone Marrow- and Adipose Tissue-derived Mesenchymal Stromal Cells are Immunosuppressive In vitro and in a Humanized Allograft Rejection Model. J. Stem Cell Res. Ther. Suppl 6, 20780. 10.4172/2157-7633.S6-001 PMC3963708 24672744

[15] Melief SM, Zwaginga JJ, Fibbe WE, Roelofs H (2013) Adipose tissue-derived multipotent stromal cells have a higher immunomodulatory capacity than their bone marrow-derived counterparts. Stem Cells Transl. Med. 2, 455–463. 10.5966/sctm.2012-0184 23694810 PMC3673757

[16] Zhang Y, Xia Y, Ni S, Gu Z, Liu H (2014) Transplantation of umbilical cord mesenchymal stem cells alleviates pneumonitis of MRL/lpr mice. J. Thorac. Dis. 6, 109–117. 10.3978/j.issn.2072-1439.2013.12.48 24605224 PMC3944187

[17] Zhou K, Zhang H, Jin O, Feng X, Yao G, Hou Y, et al (2008) Transplantation of human bone marrow mesenchymal stem cell ameliorates the autoimmune pathogenesis in MRL/lpr mice. Cell. Mol. Immunol. 5, 417–424. 10.1038/cmi.2008.52 19118507 PMC4072411

[18] Bortolotti F, Ukovich L, Razban V, Martinelli V, Ruozi G, Pelos B, et al (2015) In Vivo Therapeutic Potential of Mesenchymal Stromal Cells Depends on the Source and the Isolation Procedure. Stem Cell Reports 4, 332–339. 10.1016/j.stemcr.2015.01.001 25660405 PMC4375942

[19] Farini A, Sitzia C, Erratico S, Meregalli M, Torrente Y (2014) Clinical applications of mesenchymal stem cells in chronic diseases. Stem Cells Int. 2014, 306573 10.1155/2014/306573 24876848 PMC4021690

[20] Eggenhofer E, Benseler V, Kroemer A, Popp FC, Geissler EK, Schlitt HJ, et al (2012) Mesenchymal stem cells are short-lived and do not migrate beyond the lungs after intravenous infusion. Front. Immunol. 3, 297 10.3389/fimmu.2012.00297 23056000 PMC3458305

[21] Trivanović D, Mojsilović S, Ilić V, Krstić J, Jauković A, Okić-Đorđević I, et al (2013) Immunomodulatory capacity of human mesenchymal stem cells isolated from adipose tissue, dental pulp, peripheral blood and umbilical cord Wharton’s jelly. Cent. Eur. J. Immunol. 4, 421–429.

[22] Beyth S, Borovsky Z, Mevorach D, Liebergall M, Gazit Z, Aslan H, et al (2005) Human mesenchymal stem cells alter antigen-presenting cell maturation and induce T-cell unresponsiveness. Blood 105, 2214–2219. 15514012 10.1182/blood-2004-07-2921

[23] Gur-Wahnon D, Borovsky Z, Liebergall M, Rachmilewitz J (2009) The induction of APC with a distinct tolerogenic phenotype via contact-dependent STAT3 activation. PLoS One 4, e6846 10.1371/journal.pone.0006846 19718269 PMC2731174

[24] Németh K, Leelahavanichkul A, Yuen PST, Mayer B, Parmelee A, Doi K, et al (2009) Bone marrow stromal cells attenuate sepsis via prostaglandin E(2)-dependent reprogramming of host macrophages to increase their interleukin-10 production. Nat. Med. 15, 42–49. 10.1038/nm.1905 19098906 PMC2706487

[25] Waterman RS, Tomchuck SL, Henkle SL, Betancourt AM (2010) A new mesenchymal stem cell (MSC) paradigm: Polarization into a pro-inflammatory MSC1 or an immunosuppressive MSC2 phenotype. PLoS One 5, e10088 10.1371/journal.pone.0010088 20436665 PMC2859930

[26] Kehoe O, Cartwright A, Askari A, El Haj AJ, Middleton J (2014) Intra-articular injection of mesenchymal stem cells leads to reduced inflammation and cartilage damage in murine antigen-induced arthritis. J. Transl. Med. 12, 157 10.1186/1479-5876-12-157 24893776 PMC4053306

[27] Da Silva Meirelles L, Chagastelles PC, Nardi NB (2006) Mesenchymal stem cells reside in virtually all post-natal organs and tissues. J Cell Sci 119: 2204–2213. 16684817 10.1242/jcs.02932

[28] Kuravi SJ, McGettrick HM, Satchell SC, Saleem M A, Harper L, Williams JM, et al (2014) Podocytes regulate neutrophil recruitment by glomerular endothelial cells via IL-6-mediated crosstalk. J. Immunol. 193, 234–243. 10.4049/jimmunol.1300229 24872191 PMC4067868

[29] Lally F, Smith E, Filer A, Stone M, Shaw JS, Nash GB, et al (2005) A novel mechanism of neutrophil recruitment in a coculture model of the rheumatoid synovium. Arthritis Rheum. 52, 3460–3469. 16255036 10.1002/art.21394 PMC3119436

[30] McGettrick HM, Smith E, Filer A, Kissane S, Salmon M, Buckley CD, et al (2009) Fibroblasts from different sites may promote or inhibit recruitment of flowing lymphocytes by endothelial cells. Eur. J. Immunol. 39, 113–125. 10.1002/eji.200838232 19130557 PMC2821685

[31] Shi S, Gronthos S (2003) Perivascular niche of postnatal mesenchymal stem cells in human bone marrow and dental pulp. J Bone Miner Res 18: 696–704. 12674330 10.1359/jbmr.2003.18.4.696

[32] Pati S, Gerber MH, Menge TD, Wataha K A, Zhao Y, Baumgartner JA, et al (2011) Bone marrow derived mesenchymal stem cells inhibit inflammation and preserve vascular endothelial integrity in the lungs after hemorrhagic shock. PLoS One 6, e25171 10.1371/journal.pone.0025171 21980392 PMC3182198

[33] Munir H, Rainger GE, Nash GB, McGettrick H (2015) Analyzing the effects of stromal cells on the recruitment of leukocytes from flow. J. Vis. Exp. e52480 10.3791/52480 25590557 PMC4354496

[34] Dominici M, Le Blanc K, Mueller I, Slaper-Cortenbach I, Marini F, Krause D, et al (2006) Minimal criteria for defining multipotent mesenchymal stromal cells. The International Society for Cellular Therapy position statement. Cytotherapy 8, 315–317. 16923606 10.1080/14653240600855905

[35] Cooke BM, Usami S, Perry I, Nash GB (1993) A simplified method for culture of endothelial cells and analysis of adhesion of blood cells under conditions of flow. Microvasc. Res. 45, 33–45. 8479340 10.1006/mvre.1993.1004

[36] McGettrick HM, Buckley CD, Filer A, Rainger GE, Nash GB (2010) Stromal cells differentially regulate neutrophil and lymphocyte recruitment through the endothelium. Immunology 131, 357–370. 10.1111/j.1365-2567.2010.03307.x 20518822 PMC2992690

[37] Zhao YD, Ohkawara H, Rehman J, Wary KK, Vogel SM, Minshall RD, et al (2009) Bone marrow progenitor cells induce endothelial adherens junction integrity by sphingosine-1-phosphate-mediated Rac1 and Cdc42 signaling. Circ. Res. 105, 696–704. 10.1161/CIRCRESAHA.109.199778 19696411 PMC3402022

[38] Kaplanski G, Marin V, Montero-Julian F, Mantovani A, Farnarier C (2003) IL-6: A regulator of the transition from neutrophil to monocyte recruitment during inflammation. Trends Immunol. 24, 25–29. 12495721 10.1016/s1471-4906(02)00013-3

[39] Buron F, Perrin H, Malcus C, Héquet O, Thaunat O, Kholopp-Sarda MN, et al (2009) Human Mesenchymal Stem Cells and Immunosuppressive Drug Interactions in Allogeneic Responses: An In Vitro Study Using Human Cells. Transplant. Proc. 41, 3347–3352. 10.1016/j.transproceed.2009.08.030 19857747

[40] Nicola M Di, Carlo-stella C, Magni M, Milanesi M, Longoni PD, Grisanti S, et al (2013) Human bone marrow stromal cells suppress T-lymphocyte proliferation induced by cellular or nonspecific mitogenic stimuli. Blood 99, 3838–3843. 10.1182/blood.v99.10.3838 11986244

[41] Saeidi M, Masoud A, Shakiba Y, Hadjati J, Mohyeddin Bonab M, Nicknam MH, et al (2013) Immunomodulatory effects of human umbilical cord Wharton’s jelly-derived mesenchymal stem cells on differentiation, maturation and endocytosis of monocyte-derived dendritic cells. Iran J Allergy Asthma Immunol 12, 37–49. doi: 012.01/ijaai.3749 23454777

[42] Dalby MJ, Gadegaard N, Oreffo ROC (2014) Harnessing nanotopography and integrin-matrix interactions to influence stem cell fate. Nat Mater 13, 558–569. 10.1038/nmat3980 24845995

[43] Lee J, Abdeen AA, Kilian KA (2014) Rewiring mesenchymal stem cell lineage specification by switching the biophysical microenvironment. Sci. Rep. 4, article number 5188. 10.1038/srep05188 PMC4046125 24898422

[44] McMurray RJ, Gadegaard N, Tsimbouri PM, Burgess K V, McNamara LE, Tare R, et al (2011) Nanoscale surfaces for the long-term maintenance of mesenchymal stem cell phenotype and multipotency. Nat Mater 10, 637–644. 10.1038/nmat3058 21765399

